# Supplementary material for: The interactome of intact mitochondria by cross-linking mass spectrometry provides evidence for coexisting respiratory supercomplexes
Source: Mol Cell Proteomics. 2017 Dec 8;17(2):216–32. doi: 10.1074/mcp.RA117.000470 (PMC5795388; doi:10.1074/mcp.RA117.000470)
Supplement: Supplemental Data [file supp_RA117.000470_133922_0_supp_23978_fzffwf.zip › spectra_annotation/mito_DR_spectra_annotation/178-1-7-1-7-1.pdf]

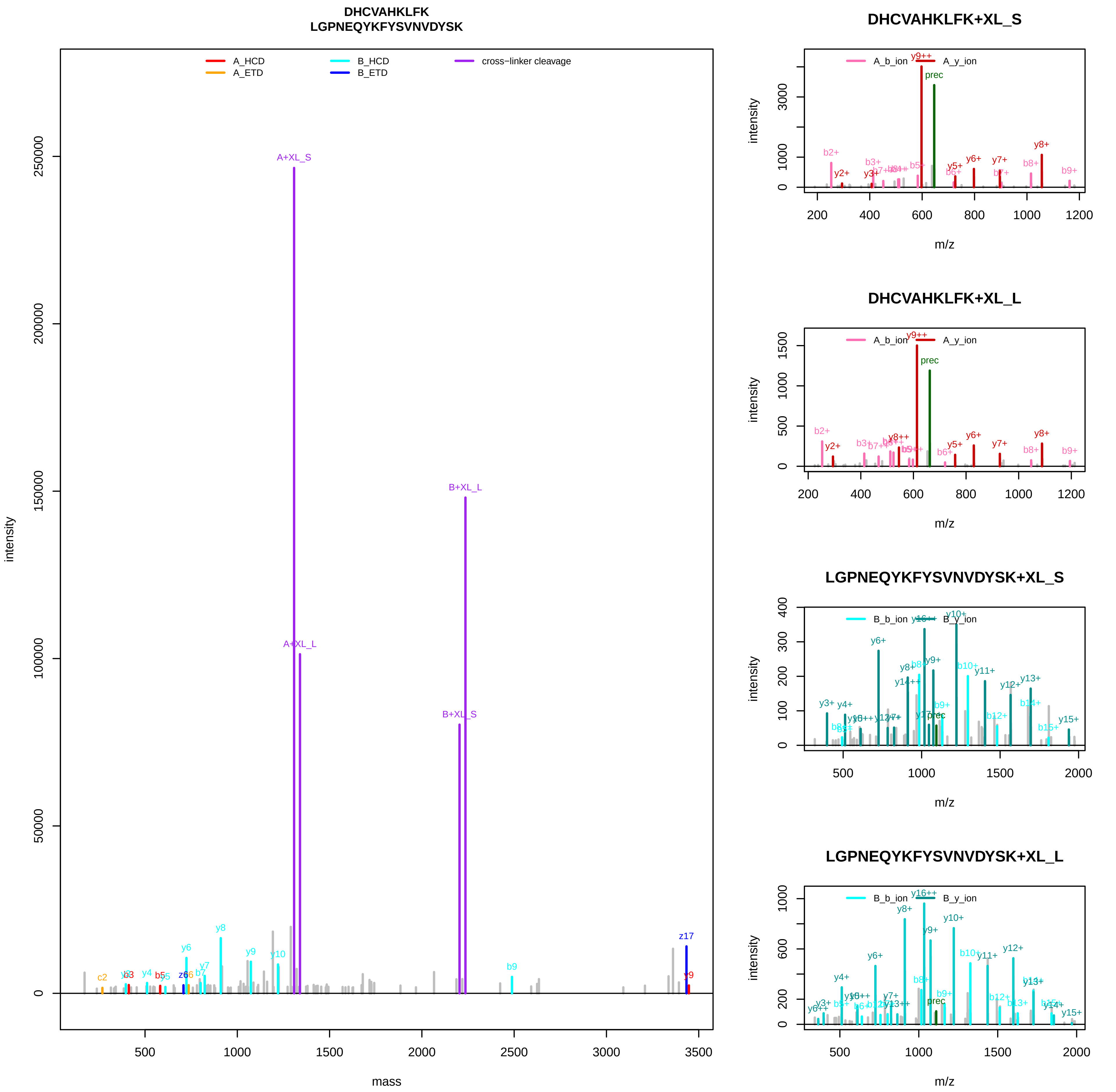

DHCVAHKLFK
LGPNEQYKFYSVNVDYSK
A_HCD
A_ETD
B_HCD
B_ETD
cross−linker cleavage
A+XL_S
A+XL_L
B+XL_L
B+XL_S
z17
y9
b9
y10
y9
y8
y7
b7
y6
z6
b5
b3
c2
y4
intensity
mass
DHCVAHKLFK+XL_S
DHCVAHKLFK+XL_L
LGPNEQYKFYSVNVDYSK+XL_S
LGPNEQYKFYSVNVDYSK+XL_L
A_b_ion
A_y_ion
B_b_ion
B_y_ion
prec
y9++
y8+
y7+
y6+
y5+
b2+
b8+
b9+
y10+
y16++
y8+
y9+
y6+
y11+
y12+
y13+
y15+
m/z
